# Eye Movements During Pareidolia: Exploring Biomarkers for Thinking and Perception Problems on the Rorschach

**DOI:** 10.3390/jemr18040032

**Published:** 2025-07-22

**Authors:** Mellisa Boyle, Barry Dauphin, Harold H. Greene, Mindee Juve, Ellen Day-Suba

**Affiliations:** 1 Department of Neurology, University of Toledo Medical Center, Toledo, OH 43606, USA; mellisa.boyle@utoledo.edu; 2 Department of Psychology, University of Detroit Mercy, Detroit, MI 48221, USA; greenehh@udmercy.edu; 3 Department of Psychology, Appleton VA Clinic, Appleton, WI 54914, USA; msjuve@gmail.com; 4 Department of Psychology, Louis Stokes Cleveland VA Medical Center, Cleveland, OH 44106, USA; ellendaysuba@gmail.com

**Keywords:** eye movements, eye-tracking, pareidolia, perception and thinking problems, Rorschach

## Abstract

Eye movements (EMs) offer valuable insights into cognitive and perceptual processes, serving as potential biomarkers for disordered thinking. This study explores the relationship between EM indices and perception and thinking problems in the Rorschach Performance Assessment System (R-PAS). Sixty non-clinical participants underwent eye-tracking while completing the Rorschach test, focusing on variables from the Perception and Thinking Problems Domain (e.g., WSumCog, SevCog, FQo%). The results reveal that increased cognitive disturbances were associated with greater exploratory activity but reduced processing efficiency. Regression analyses highlighted the strong predictive role of cognitive variables (e.g., WSumCog) over perceptual ones (e.g., FQo%). Minimal overlap was observed between performance-based (R-PAS) and self-report measures (BSI), underscoring the need for multi-method approaches. The findings suggest that EM patterns could serve as biomarkers for early detection and intervention, offering a foundation for future research on psychotic-spectrum processes in clinical and non-clinical populations.

## 1. Introduction

Eye movements (EMs) are promising behavioral correlates of cognitive, social, and emotional functioning as they are sophisticated processes controlled by the brain, and due to their low cost, non-invasive nature, and objectivity in measurement (Liversedge et al.) [1]. When responding to the Rorschach, individuals visually scan blots to identify features that align with familiar objects, essentially constructing recognizable objects from the variations in shading and form. This is commonly known as the process of pareidolia (Sibbald et al.) [2].

Recent studies by Ales et al. [3] and Dauphin et al. [4] have shown the value of EMs in understanding processing strategies during the Rorschach by establishing associations between EM indices and variables from the Rorschach Performance Assessment System (R-PAS) (Meyer et al.) [5]. These studies found that participants with higher Complexity scores exhibited more fixations while scanning the blots, indicating higher levels of cognitive engagement, consistent with the R-PAS interpretation of complexity. Additionally, participants showed shorter fixation durations as their Vague Percent (Vg%) increased, suggesting shallower information processing. These findings demonstrate the usefulness of R-PAS variables as correlates to EMs for understanding how pareidolic processing strategies are associated with implicit personality characteristics in the Engagement and Cognitive Processing Domain (Meyer et al.) [5].

The current study aims to explore eye movement correlates of the Perception and Thinking Problems Domain of the R-PAS [5], which largely represents issues in thinking, judgment, or perception. Variables such as Fo%, POP codes, WSumCog, SevCog, EII-3, and TP-Comp are related to conventionality, reality testing, thought disorganization, and disturbing thought content, involving both thinking and perceptual aspects (Meyer et al.) [5]. The goal is to understand options for detecting disordered thinking and the utility of methods for doing so. While some research has investigated features of disordered thinking in non-clinical individuals, most attention has been paid to clinically diagnosed individuals. Using non-clinical populations can be valuable, as many current models propose a continuum of psychotic experiences in the population (DeRosse et al. [6], vanOs et al. [7], and Verdoux and vanOs) [8]. Investigating non-clinical populations can also contribute to using EMs for early detection and prevention and improving the specificity of EMs as biomarkers of psychosis. This exploratory study aims to establish strong behavioral biomarker candidates for predictive hypotheses in future studies of clinical and non-clinical samples.

## 2. Materials and Methods

### 2.1. Participants

Sixty individuals (18 males) from a Midwestern University’s undergraduate participant pool and the surrounding community participated in this study (age range: 18 to 60 years, mean = 25.73, S.D. = 8.72). All participants had normal or corrected-to-normal visual acuity, and color blindness was an exclusion criterion. Participants were paid USD 30 for their time in the laboratory (2–2 1/2 h). This study was approved by the university’s Institutional Review Board (Protocol #1314-41) and conducted in accordance with the Belmont Principles (1978), with all participants providing informed consent.

### 2.2. Stimuli

Ten Rorschach inkblots (Exner) [9] were digitized at a resolution of 150 DPI. At viewing distances in the range of 50–70 cm (i.e., scanned image areas between 21° × 18° and 15° × 13°), and with ambient lighting between 40 and 200 Lux near the eye-tracking station, scanned images (on a 70 cd/m^2^ background) and card images were judged to be metameric by five individuals in the laboratory.

### 2.3. Apparatus

Light levels near the eye-tracking station were measured by a SPER SCIENTIFIC 840006 light meter (SPER SCIENTIFIC LTD, Scottsdale, AZ, USA). Background luminance on the color monitor was measured by a Konica Minolta LS-110 luminance meter, Ramsey, NJ, USA). The digitized Rorschach images were presented on a 17-inch (i.e., 43-cm) color monitor (26 cm × 34 cm, 800 × 600 pixels, 75 Hz refresh rate). Eye movement characteristics were recorded using an Eyelink-II Eye-Tracking system with head-mounted video cameras tracking corneal reflection in combination with pupil position at 500 Hz (SR Research, Toronto, Ontario, Canada) as participants viewed the Rorschach images. An eye movement was detected when the eye velocity exceeded 30° s^−1^ or eye acceleration exceeded 8000° s^−2^. After a 9-point calibration, the reported gaze-position accuracy for each participant was in the visual angle range of 0.5–1.0°. Eyelink II can maintain this accuracy despite minor head movements less than a visual angle of ±15° (e.g., from uttering verbal responses during the Rorschach test). Stimulus presentation and eye-tracking were controlled by University of Massachusetts EyeTrack 7.9F software (see https://websites.umass.edu/eyelab/software/ (accessed on 4 July 2024) for updated versions of this software).

### 2.4. Measures

Eye movement indices used in this study and their definitions are provided in Table 1. The focus of the current study is the R-PAS Perception and Thinking Problems Domain. Page 1 and Page 2 variables and brief descriptions of their interpretive significance can be found in Table 2.

In addition, the Brief Symptom Inventory (Derogatis) [10] was administered to the participants. The scales of Paranoid Ideation (Pa), Psychoticism (Py), Global Severity Index (GSI), Positive Symptom Index (PSI), and the Positive Symptom Distress Index (PSDI) were the focus of analyses to understand perception and thinking problems and compare self-report measures with performance-based measures.

### 2.5. Procedure

Participants completed a demographic questionnaire, self-report measures, and a performance-based measure (not in the scope of the present study). Eyelink II eye-tracking cameras were fitted for comfort during extended use. Participants sat 55 cm to 65 cm from the monitor, with Rorschach images subtending visual angles of approximately 17° by 15°. Viewing was binocular, but recording was monocular, measuring only right-eye movements [1]. Binocular recordings of eye movements is only of concern during some reading studies where a very fine spatial resolution is of importance (e.g., Liversedge et al., [11,12]), and do not outweigh the set-up convenience and cost-savings to researchers or the comfort of participants offered by monocular recordings of eye movements. The eye-tracker was set to perform an eye drift before each presentation of a Rorschach image to maintain eye-tracking accuracy.

The R-PAS administration of the Rorschach (Meyer et al.) [5] was utilized with minor modifications to account for viewing the images on a computer screen. Modified R-PAS instructions were: Okay, now we are ready to start. I will show the inkblots to you one at a time. Your task is to look at each inkblot to answer the question ‘What might this be?’ Does that make sense? YES: Good, we can get started then. Try to give two responses…or maybe three, to each inkblot. That is, for each inkblot try to see two different things; possibly three. Let me know when you have finished responding, so we can go to the next inkblot. (DISPLAY CARD 1). What might this be?

The images were presented sequentially (Cards I-X), and a trial was terminated when the participant indicated completion. The Clarification phase was completed with physical cards, according to the standardized R-PAS procedure, after removing the eye-tracker. During this phase, location areas for scoring (e.g., Whole, Common Detail) were determined by the examiner based on the participant’s verbal and physical indications on the cards; these R-PAS location codes were methodologically distinct from the grid-based URV coordinates collected via eye-tracking during the Response Phase. Rorschach verbal responses were coded and scored according to R-PAS criteria (Meyer et al.) [5].

Participants provided verbal responses while viewing the digitized inkblots on the screen during the Response Phase, with the eye-tracker recording their visual exploration patterns. This approach enabled the simultaneous capture of both verbal responses and corresponding eye movements. While this methodology does not differentiate between fixations related to perceptual encoding versus those driven by verbal planning or decision processes (Laubrock & Klieg [13]; Johansson & Johansson [14]; Matsumiya & Furukawa) [15], it captures naturalistic visual exploration during the complete response process. Thus, eye-tracking data were collected exclusively during the Response Phase and not during the subsequent Clarification Phase. The eye-tracker was removed prior to the Clarification Phase to enhance participant comfort during this extended interaction with the physical cards.

Each participant’s responses were coded by the second and fourth authors to consensus, both of whom had extensive experience in R-PAS training, teaching, and supervision of students learning the R-PAS. Following the initial scoring, the authors met to compare their scores and resolve any discrepancies through a discussion and reference to the R-PAS manual. This collaborative process allowed the scorers to share their rationales, ensuring a thorough examination of each protocol and a comprehensive understanding of the scoring criteria. The consensus scoring approach was chosen to ensure the highest level of accuracy and consistency in the scoring of the Rorschach protocols.

## 3. Results

### 3.1. R-PAS and BSI

The first aspect to consider is the correspondence between the performance measures (R-PAS) and self-report measures (BSI). Table 3 contains the Descriptive Statistics for the variables in this study, while Table 4 illustrates the Pearson correlations between these two sets of variables measuring disturbances of thinking and reality testing. Psychoticism was inversely correlated with FQo%, indicating that higher Psychoticism scores are associated with lower levels of ordinary form quality on the Rorschach. The GSI was correlated with FQu%, suggesting that a higher Global Severity of self-reported symptoms was related to more unusual form quality in the participant’s Rorschach protocol. No other relationships were statistically significant.

Next, we examine the relationships between the EM variables, R-PAS variables, and BSI variables in Table 4. Overall, there were several significant correlations between R-PAS variables of Perception and Thinking Problems and NF, URV, and URV/NF. NF, URV, and URV/sec were correlated with EII-3, TP_Comp, WSumCog, Sev Cog, and FQo%. URVV/NF was correlated with EII-3, TP_Comp, WSumCog, and Sev Cog. In contrast, there were several significant correlations between BSI variables and FD (Pa, PY, GSI, and PSI), indicating that the two different methods of assessing psychotic or psychotic-spectrum processes are related to different facets of the allocation of attention and information processing. Please see the Supplemental Materials for an example of EM scanpath for a Rorschach card, card level histograms of viewing duration, and for visual examples of the relationship between select EM and R-PAS variables (Figures S1–S3).

### 3.2. Cognitive and Perceptual Variables

Given the relationships between several R-PAS variables and EM indices, it is useful to gain further insights into the findings. The Perception and Thinking Problems Domain contains variables that are more sensitive to perception (e.g., FQo%, FQu%, FQ-%) and others more sensitive to thinking or cognitive aspects (e.g., WSumCog and SevCog). To address whether the variation associated with EMs can be accounted for by perceptually relevant variables, cognitively relevant variables, or both simple regressions were run with each of the significant EM variables (i.e., NF, URV, URV/NF). A perceptually related variable (FQo%) and a cognitively related variable (WSumCog) were selected as predictor variables.

The first regression analysis revealed the relationship between the dependent variable, NF, and two predictors: WSumCog and FQo%. The model summary indicated a R^2^ of 0.171, suggesting that approximately 17.1% of the variance in NF was explained by the predictors. The adjusted R^2^ value was slightly lower at 0.142, accounting for the number of predictors in the model. The F-statistic for the change in R^2^ was significant F (2, 57) = 5.880, *p* = 0.005, indicating that the model significantly predicts the dependent variable.

The coefficients table shows that WSumCog has a positive relationship with NF (B = 9.657, *p* = 0.010), whereas FQo% shows a negative relationship, though not statistically significant (B = −10.808, *p* = 0.131). The constant term is significantly different from zero (B = 1638.297, *p* < 0.001). Table 5 summarizes the regression analysis.

The second regression analysis shows how the dependent variable URV was influenced by two predictors: WSumCog and FQo%. The model summary revealed an R^2^ value of 0.148, indicating that about 14.8% of the variance in URV was explained by these predictors. The adjusted R^2^ is 0.119, which adjusts for the number of predictors. The model’s change in R^2^ was statistically significant F (2, 57) = 4.969, *p* = 0.010, affirming that the model is a good fit for the data.

From the coefficients table, WSumCog exhibits a positive association with URV (B = 1.296, *p* = 0.043), while FQo% shows a negative association, which is not statistically significant (B = −2.259, *p* = 0.068). The intercept was significantly different from zero (B = 462.253, *p* < 0.001). Table 6 summarizes the regression analysis.

The third regression analysis examined the relationship between the dependent variable URV/NF and two predictors: WSumCog and FQo%. The model summary showed a R^2^ of 0.116, indicating that approximately 11.6% of the variance in the dependent variable is explained by these predictors. The adjusted R^2^ was lower at 0.085, reflecting the adjustment for the number of predictors. The model’s R^2^ change was statistically significant F (2, 57) = 3.731, *p* = 0.030, suggesting that the model provides a significant fit to the data.

In the coefficients table, WSumCog is negatively associated with the dependent variable (B = −0.001, *p* = 0.042), while FQo% shows a positive but not statistically significant association (B = 0.001, *p* = 0.211). The intercept was significantly different from zero (B = 0.281, *p* < 0.001). Table 7 summarizes the regression analysis.

The fourth regression analysis examined the relationship between the dependent variable URV/sec and two predictors: WSumCog and FQo%. The model achieved a R^2^ of 0.112, suggesting that approximately 11.2% of the variance in the dependent variable was explained by these predictors. The adjusted R^2^ lowers slightly to 0.081, considering the number of predictors. The model’s change in R^2^ was statistically significant F (2, 57) = 3.605, *p* = 0.034), indicating a significant predictive capability. Table 8 summarizes the regression analysis.

From the coefficients table, WSumCog shows a negative association with URV/sec (B = −0.002, *p* = 0.109), although this effect is not statistically significant. FQo% exhibits a positive association with URV/sec (B = 0.005, *p* = 0.090), also not reaching statistical significance. Table 9 summarizes the regression analysis.

## 4. Discussion

The results show several significant relationships between R-PAS variables and EM indices, suggesting different levels of pareidolic processing for individuals experiencing varying levels of thinking and perception problems. Both cognitive and perceptual variables indicate that increasing levels of difficulty are associated with more fixations and visiting more regions of the inkblots, suggesting a more exhaustive and possibly scattered search pattern. This pattern could indicate a higher cognitive load (Rayner) [16,17], as individuals struggle to make sense of the stimuli, potentially reflecting their efforts to impose structure or find meaning in ambiguous situations. At the same time, higher degrees of thinking disturbance are associated with generally less efficient processing, as higher levels of scores for EII-3, TP_Comp, WSumCog, and FQ-% are inversely correlated to URV/NF. The increased cognitive load could lead to less efficient processing for individuals with greater difficulties in thinking and reality testing, as they might revisit the same concepts or struggle with integrating information cohesively.

The regression analyses indicated that the cognitive variables likely contribute more to the variance compared to the perceptual variables. WSumCog, which measures thinking disturbance, predicted a significant amount of variance for EM indices of NF, URV, and URV/NF, while FQo% failed to reach significance. As thinking disturbance increases, participants showed greater exploratory activity but exhibited less efficient processing, suggesting that participants with higher degrees of thinking disturbance might utilize more effort to perform the task. It is possible that even subclinical thinking disturbances could lead to this loss of efficiency.

The positive correlations between Perception and Thinking Problems variables (EII-3, TP-Comp, WSumCog, SevCog) and exploratory EMs (NF, URV) warrant careful interpretation. While these patterns align with the cognitive dysmetria framework proposed for psychotic disorders (Andreasen et al.) [18], alternative explanations must be considered. The increased gaze dispersion observed could represent cognitive disorganization or, alternatively, adaptive exploratory search strategies employed to compensate for subtle perceptual integration challenges. Individuals with higher Perception and Thinking Problems scores may adopt more comprehensive scanning behaviors to offset processing inefficiencies. However, the negative relationship between these variables and URV/NF (an efficiency metric) suggests that despite increased exploration, information extraction per fixation decreases—a finding more consistent with the reduced processing model of psychosis spectrum disorders (Phillips & Silverstein) [19] than with purely strategic exploration. These statistically significant relationships indicate a reliable association between cognitive integration abilities and visual processing efficiency that may exist on a continuum, even in non-clinical populations. Future research employing experimental manipulations of task demands could help differentiate between these competing explanations by examining whether gaze dispersion patterns persist across different cognitive contexts.

There was very little overlap between R-PAS and BSI, with a small degree of convergence between self-report and performance measures, consistent with the research. This finding argues for the use of multiple methods in the research.

The current study’s findings have several implications for understanding the relationship between eye movements, pareidolic perception, and thinking problems. Eye movements have been used as behavioral biomarkers for disordered thinking in schizophrenia, with a well-established body of literature documenting abnormalities in individuals with schizophrenia diagnoses (Miassian [20]; Phillips & Silverstein [19]; Silverstein et al. [21]; Yoon et al. [22]). Eye movement methodology can help overcome methodological issues related to generalized performance deficits in perception and thinking disturbances by measuring task performance variability across group comparisons, manipulating visual stimuli independently of cognitive demands, and controlling for differences in stimulus detection and orientation (Yoon et al.) [22]. This research aimed to establish biophysiologic models of cognition (Daskalakis) [23] and provide information for future clinical and research applications. Understanding how eye movement behavior varies with stimulus type, task, and features of information processing disturbance is crucial for elucidating the clinical relevance of eye movement behavior as a biomarker for disordered thinking.

The present study focused on extending knowledge of relationships between EMs as bio-behavioral markers and cognitive dysfunctions seen in psychotic disorders, particularly in non-clinical individuals who, to varying degrees, report or perform in ways that deviate from typical perception and thinking. The results suggest that eye movement indices can serve as valuable bio-behavioral markers for detecting and characterizing cognitive dysfunctions associated with psychotic disorders, even in non-clinical populations. The use of eye-tracking technology in conjunction with the Rorschach test may provide a more comprehensive assessment of an individual’s cognitive and perceptual functioning, potentially aiding in early detection and prevention efforts.

Furthermore, this study highlights the importance of considering both cognitive and perceptual aspects when investigating thinking and perception problems. The findings indicate that cognitive variables, such as thinking disturbance measured by WSumCog, may have a greater influence on eye movement patterns compared to perceptual variables. This insight can guide future research efforts and inform the development of targeted interventions for individuals experiencing cognitive difficulties.

### Limitations and Future Directions

While the current study provides valuable insights, it is important to acknowledge its limitations. While participants could not rotate the digital images during the Response Phase as they could with physical cards during Clarification, this limitation was deemed an acceptable tradeoff to obtain precise eye movement data during initial response formulation. Additionally, our sample size of 60 participants, while adequate for the linear regression analyses employed, would likely be insufficient for more complex approaches such as Generalized Additive Models. Future research with larger samples could explore potential nonlinear relationships that might not be captured by our current analyses. Future research should aim to replicate these results with larger and more diverse samples, including both clinical and non-clinical populations.

This study relied on a single performance-based measure (Rorschach) and a single self-report measure (BSI) to assess perception and thinking problems. Incorporating a broader range of assessment tools, such as cognitive tasks and neuroimaging techniques, could provide a more comprehensive understanding of the relationship between eye movements and cognitive dysfunctions.

Future research should also explore the potential of using eye movement indices as predictive markers for the development of psychotic disorders. Longitudinal studies following individuals with subclinical thinking disturbances could help establish the predictive value of eye movement patterns in identifying those at risk for developing more severe psychopathology.

Moreover, investigating the neural correlates of eye movement abnormalities in individuals with perception and thinking problems could provide valuable insights into the underlying mechanisms of these dysfunctions. Combining eye-tracking with neuroimaging techniques, such as functional magnetic resonance imaging (fMRI; e.g., Akdeniz, et al. [24]) or electroencephalography (EEG) (Cyders, M.A.; Coskunpinar [25]), could help elucidate the brain regions and networks involved in aberrant cognitive processing.

A methodological consideration when interpreting our findings involves the digital presentation of Rorschach stimuli compared to traditional card administration. This potential concern was empirically addressed by Ales et al. [26], who examined the remote digital administration of the Rorschach, finding that complexity scores—the primary measure of engagement with the task—showed no significant differences from normative expectations derived from standard in-person procedures (d = 0.055). While their exploratory analyses indicated some differences in specific variables, the fundamental comparability of core engagement metrics across administration formats supports the validity of our methodology. This evidence suggests that the digital presentation of stimuli likely preserves the essential perceptual and cognitive processes measured by the Rorschach, though continued research on equivalence across all response dimensions remains important.

In conclusion, the current study demonstrates the value of using eye movement indices as bio-behavioral markers for understanding perception and thinking problems in non-clinical populations. The findings highlight the importance of considering both cognitive and perceptual aspects when investigating these dysfunctions and provide a foundation for future research aimed at improving early detection, prevention, and intervention efforts for individuals at risk of developing psychotic disorders.

## Figures and Tables

**Table 1:** Definitions of eye-tracking variables used in the present study.

Eye-Tracking Measure	Definition
NF	The number of fixations used to scan the images.
FD	The mean duration of the time the eye is still while a participant acquires information from these images.
SA	The mean distance the eye travels in search of information.
ISA	The eye distance traveled at the onset of each image.
ISL	The reaction time at the onset of each image.
URV	Method of dividing the stimulus field into non-overlapping regions of equal size, which receive at least one fixation, based upon a virtual 12 × 16 grid overlay of the display screen during data analysis. With this definition, each URV square subtended a visual area of 2° × 2°.
URV/NF	Number of unique regions visited per number of fixations.
NF/sec	Number of fixations per second.
URV/sec	Number of unique regions visited per second.

**Table 2:** Page 1 and Page 2 R-PAS variables from the Perception and Thinking Problems Domain.

Variable	Name	Brief Interpretation
Page 1		
EII-3	Ego Impairment Index	Thinking disturbance and how severe the psychopathology (if present) is.
TP-Comp	Thought and Perception Composite	Reality testing; disorganized thinking.
WSumCog	Weighted Sum of the Six Cognitive Codes	Disordered and disturbing thinking.
SevCog	Severe Cognitive Codes	Evidence of severe problems with thinking.
FQ–%	Form Quality—%	Distortions of reality; not perceiving the world as others do.
WD–%	Percent of W’s and D’s that have Minus Form Quality	Distortions of reality are present, even in conventional or common situations.
FQo%	Ordinary Form Quality Percentage	Seeing the world in the ways that others do.
P	Popular	Seeing the world in the ways others do, but also in highly conventional ways.
Page 2		
FQu%	Unusual Form Quality Percentage	Seeing the world in a somewhat different way than others do and, as a result, having a tendency to engage in more individualistic behaviors.

**Table 3:** Descriptive statistics for eye-tracking variables, R-PAS Perception and Thinking Problems, and BSI.

Descriptive Statistics					
	N	Minimum	Maximum	Mean	SD
NF	60	40.30	290.00	133.95	63.51
FD	60	186.89	468.08	331.09	51.75
SA	60	1.47	4.06	2.62	0.52
URV	60	20.40	70.30	38.04	10.78
ISA	60	0.86	3.40	1.92	0.60
ISL	60	176.70	613.70	280.48	71.30
URV/NF	60	0.14	0.51	0.32	0.086
NF/sec	60	1.26	3.66	2.52	0.423
URV/sec	60	0.35	1.33	0.79	0.24
EII_3	60	−1.50	3.40	0.87	1.01
TP_Comp	60	−0.90	3.50	1.43	0.88
WSumCog	60	0.00	88.00	26.90	21.60
SevCog	60	0.00	9.00	1.92	2.18
FQo	60	5.00	17.00	12.15	2.43
FQm	60	0.00	7.00	3.35	1.79
WDm	60	0.00	6.00	2.12	1.34
FQu	60	2.00	17.00	8.18	2.96
BSI_PA	60	41.00	80.00	52.72	10.57
BSI_PY	60	45.00	80.00	52.65	10.16
BSI_GSI	60	32.00	72.00	49.95	10.79
BSI_PST	60	29.00	78.00	50.55	11.24
BSI_PSDI	60	29.00	67.00	50.47	8.165

**Table 4:** Intercorrelations between Perception and Thinking Problems Domain and BSI variables.

Perception and Thinking Problems	Paranoid Ideation	Psychoticism	GSI	PST	PSDI
Page 1					
EII_3	0.053	0.113	−0.038	−0.046	0.012
TP_Comp	0.010	0.150	−0.030	−0.035	0.038
WSumCog	−0.002	0.091	−0.006	−0.017	0.057
SevCog	0.001	0.115	0.006	0.001	0.100
FQm_Per	0.062	0.122	−0.023	−0.008	−0.059
WDm_Per	0.095	0.090	0.087	0.063	0.124
FQo_Per	−0.146	−0.312 *	−0.233	−0.231	−0.121
Popular	−0.032	−0.020	−0.100	−0.085	0.017
Page 2					
FQu%	0.121	0.226	0.270 *	0.252	0.173

* *p* < 0.05 GSI (Global Severity Index); PST (Positive Symptom Total); PSDI (Positive Symptom Distress Index).

**Table 5:** Correlations between EM variables; R-PAS Thinking and Perception Problems Domain.

Eye-Tracking	EII_3	TPComp	WSumCog	SevCog	FQm%	WDm%	FQo%	P	FQu% ^+^	BSIPA	BSIPY	BSIGSI	BSIPST	BSIPSDI
NF	0.455 **	0.401 **	0.370 **	0.310 *	0.202	0.120	−0.261 *	−0.110	0.160	−0.004	−0.180	−0.114	−0.140	−0.093
FD	0.027	0.065	−0.084	−0.087	0.175	0.178	0.007	−0.145	−0.121	−0.260 *	−0.264 *	−0.255 *	−0.268 *	−0.228
URV	0.337 **	0.313 *	0.311 *	0.261 *	0.177	0.109	−0.290 *	−0.144	0.190	0.026	−0.094	−0.051	−0.070	0.002
SA	−0.008	0.030	0.039	0.002	0.070	0.216	−0.221	−0.125	0.179	−0.005	0.086	−0.028	−0.034	−0.106
URV/NF	−0.395 **	−0.319 *	−0.302 *	−0.249	−0.115	−0.050	0.220	0.035	−0.197	−0.071	0.150	0.017	0.034	0.159
NF/sec	−0.004	−0.119	0.032	0.022	−0.222	−0.262 *	0.088	0.290 *	0.058	0.080	0.071	0.138	0.145	0.063
URV/sec	−0.370 **	−0.392 **	−0.257 *	−0.208	−0.267 *	−0.235	0.267 *	0.227	0.140	−0.011	0.177	0.096	0.115	0.175

* *p* < 0.05; ** *p* < 0.01; ^+^ Page 2 variable.

**Table 6:** Regression analysis for the dependent variable of NF and independent variables of WSumCog and FQ0Per.

Predictor	B	SE	β	t	*p*
Constant	1638.297	404.964		40.046	<0.001
WSumCog	9.657	3.635	0.328	2.657	0.010
FQo_Per	−10.808	70.056	−0.189	−1.532	0.131

**Table 7:** Regression analysis for the dependent variable of URV and independent variables of WSumCog and FQ0Per.

Predictor	B	SE	β	t	*p*
Constant	462.253	69.643		6.637	<0.001
WSumCog	1.296	0.625	0.260	2.073	0.043
FQo_Per	−2.259	1.214	−0.233	−1.861	0.068

**Table 8:** Regression analysis for the dependent variable of URV/NF and independent variables of WSumCog and FQoPer.

Predictor	B	SE	β	t	*p*
Constant	0.281	0.057		4.962	<0.001
WSumCog	−0.001	0.001	−0.266	−2.084	0.042
FQo_Per	0.001	0.001	0.162	1.265	0.211

**Table 9:** Regression analysis for the dependent variable of URV/sec and independent variables of WSumCog and FQo%.

Predictor	B	SE	β	t	*p*
Constant	0.610	0.155		3.925	<0.001
WSumCog	−0.002	0.001	−0.208	−1.627	0.109
FQo_Per	0.005	0.003	0.221	1.726	0.090

## Data Availability

The original data presented in this study are available at: https://udmercy0-my.sharepoint.com/:f:/g/personal/dauphivb_udmercy_edu/Ei9xBumAFi1LjDBYwjTpRjYB7zgrr9YOhQTC8tNKgFoDEw?e=u6ikQf (accessed on 4 July 2025).

## Supplementary Materials

The following supporting information can be downloaded at: https://www.mdpi.com/article/10.3390/jemr18040032/s1, Figure S1: Example of a scanpath during viewing (Card IV); Figure S2: Histograms of participant durations (msec) for responding for the 10 Rorschach blots; Figure S3: Examples of the relationships between EMs and select R-PAS Perception and Thinking Problems variables.
