# Supplementary material for: Eye Movements During Pareidolia: Exploring Biomarkers for Thinking and Perception Problems on the Rorschach
Source: J Eye Mov Res. 2025 Jul 22;18(4):32. doi: 10.3390/jemr18040032 (PMC12387255; doi:10.3390/jemr18040032)
Supplement: Supplementary file 1 [file jemr-18-00032-s001.zip › jemr-3594820-supplementary/Figures JEMR/Figure 3.pdf]

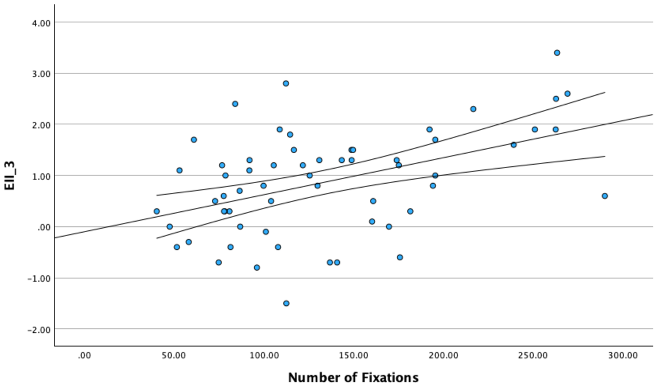


Figure S3a. Scatterdot relationship between Number of Fixations and EII-3

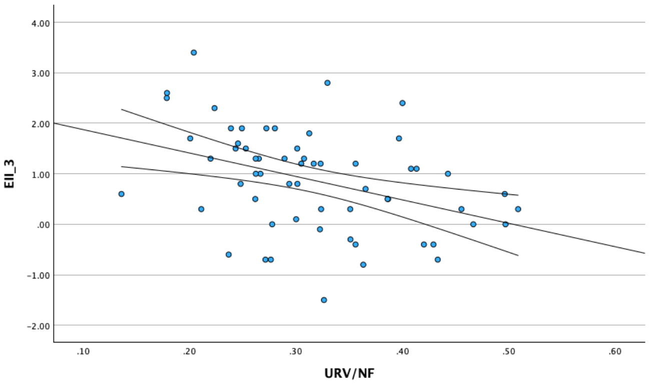


Figure S3b. Scatterdot Relationship between URV/NF and EII-3

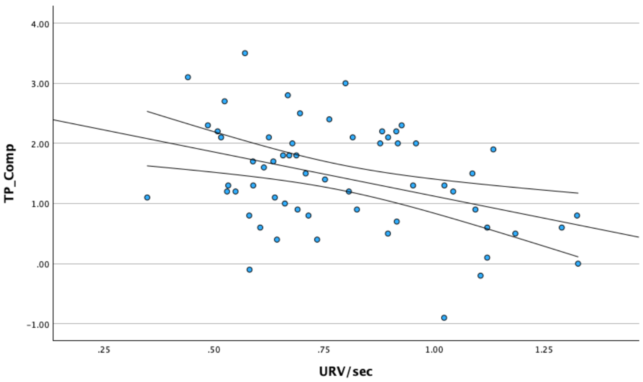


Figure S3c. Scatterdot relationship between URV per second and TP-COMP

Figure S3. Examples of the relationships between EMs and select R-PAS Pe rception and Thinking Problems variables.
