# Supplementary figures and images for: Eye Movements During Pareidolia: Exploring Biomarkers for Thinking and Perception Problems on the Rorschach
Source: J Eye Mov Res. 2025 Jul 22;18(4):32. doi: 10.3390/jemr18040032 (PMC12387255; doi:10.3390/jemr18040032)

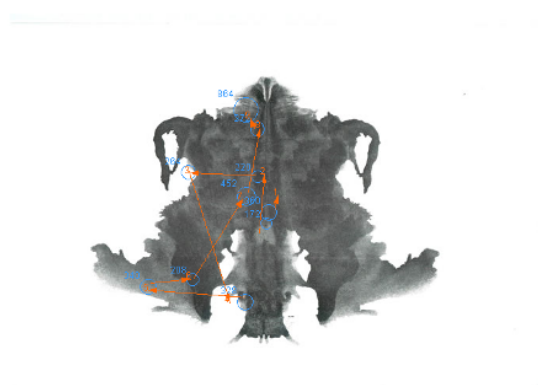

Figure S1. Example of a scanpath during viewing (Card IV)

Supplement: Supplementary file 1 [file jemr-18-00032-s001.zip › jemr-3594820-supplementary/Figures JEMR/Figure 1.pdf]

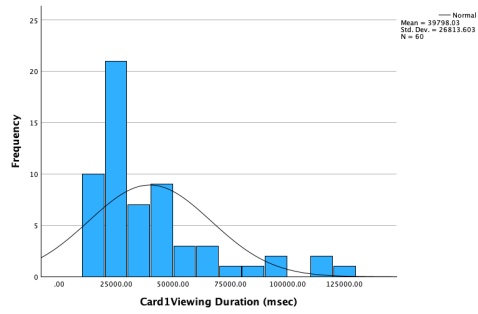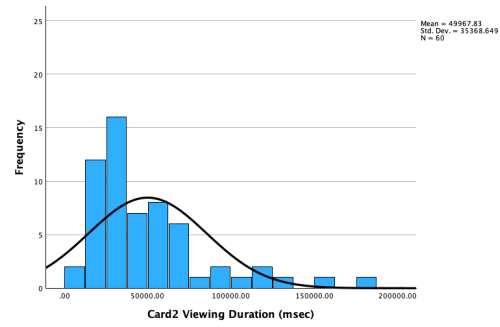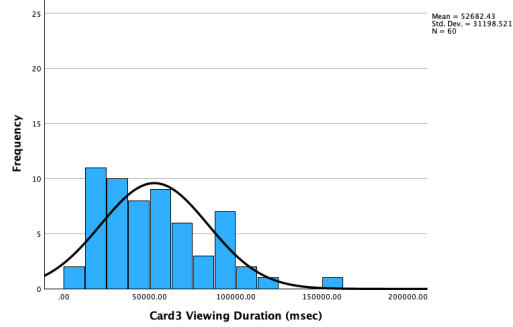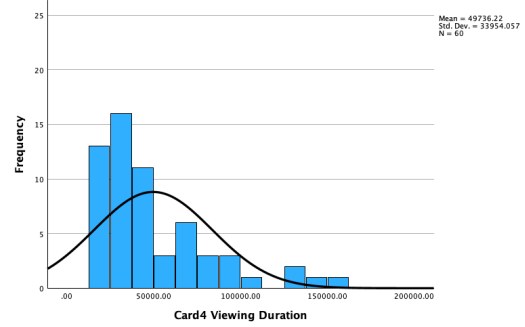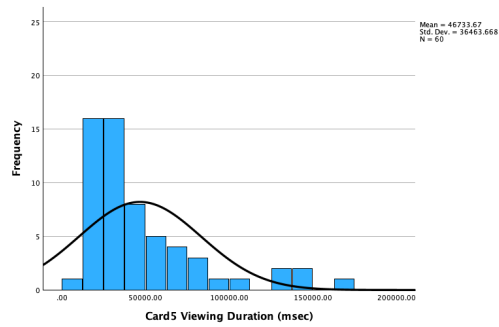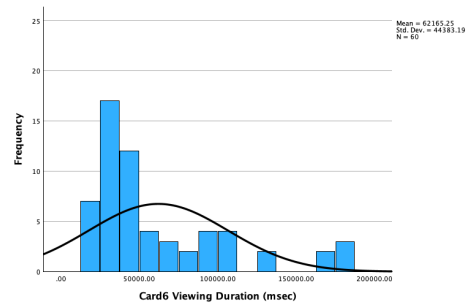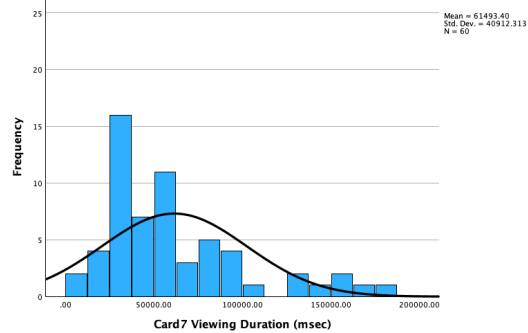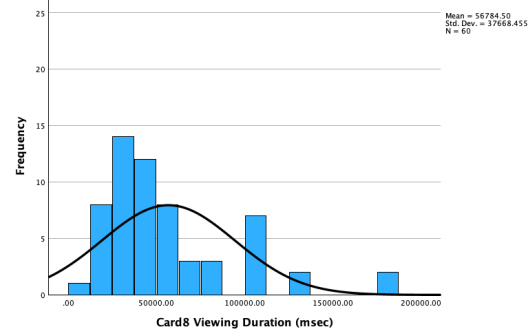

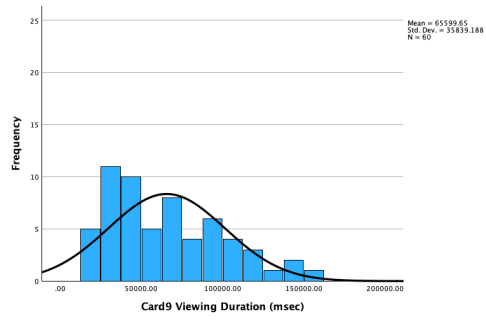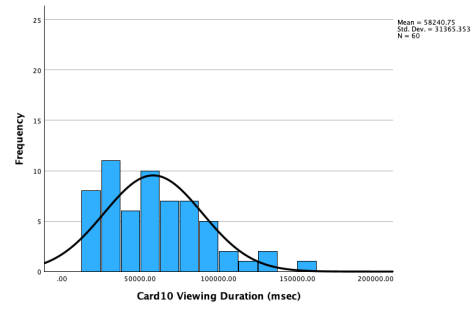

Figure S2. Histograms of participant durations (msec) for responding for the 10 Rorschach blots.

Supplement: Supplementary file 1 [file jemr-18-00032-s001.zip › jemr-3594820-supplementary/Figures JEMR/Figure 2.pdf]
